# Bioremediation of Crude Oil by Rhizosphere Fungal Isolates in the Presence of Silver Nanoparticles

**DOI:** 10.3390/ijerph17186564

**Published:** 2020-09-09

**Authors:** Mayasar I. Al-Zaban, Mohamed A. Mahmoud, Maha A. AlHarbi, Aisha M. Bahatheq

**Affiliations:** 1 Biology Department, College of Science, Princess Nourah Bint Abdulrahman University, Riyadh 11671, Saudi Arabia; mialzaban@pnu.edu.sa (M.I.A.-Z.); maalharbi@pnu.edu.sa (M.A.A.); amb.sa07@hotmail.com (A.M.B.); 2 Molecular Markers Laboratory, Plant Pathology Research Institute, Agricultural Research Center, Giza 12619, Egypt

**Keywords:** biodegradation, crude oil, fungi, nanoparticles, response surface method, ISSR marker

## Abstract

Background: This research work focuses on the utilization of indigenous fungi for in situ bioremediation of crude oil in the presence of silver nanoparticles. Methods: Two fungi belonging to two different genera showed promising crude oil-degrading abilities. Fungal isolates were identified based on internal transcribed spacer rDNA sequence analysis. Gas chromatography-mass spectrometry analysis of the crude oil remaining in the culture medium after seven days was performed. The response surface method (RSM) designed by Box-Behnken was used to establish a mathematical model. Inter-simple sequence repeat (ISSR) primers were used to examine the genetic variation of fungal isolates. Results: Gas chromatography-mass spectrometry (GC-MS) analysis after seven days showed that the optimum biodegradation of crude oil was 57.8%. The crude oil degradation rate was significantly affected by a temperature of 30 °C, pH value of 7, crude oil concentration of 4 g/L, a 1:1 ratio between *A. flavus* AF15 and *T. harzianum* TH07, and an silver nanoparticle (AgNP) concentration of 0.05 g. Molecular characterization in fungal isolates is extremely valuable when using ISSR markers. Conclusions: Two fungal isolates showed promising crude oil-degrading abilities with positive effect of low concentrations of AgNPs on biodegradation. RSM is an efficient mathematical method to optimize the microbial biodegradation of crude oil.

## 1. Introduction

Saudi Arabia, which has almost a quarter of the world’s oil reserves, is the largest producer and exporter of oil. Petroleum is transported around the world via pipelines, roads, ships, and trains, posing a significant environmental threat in the event of spills. Crude oil is an extremely toxic carcinogenic, is strongly mutagenic, and consists of teratogenic complex compounds. It is recognized as a serious threat to ecosystems and it takes several years or decades to recover from many environmental problems after the event of a spill [1]. The presence of crude oil in the natural environment is a critical problem because it causes gradual soil degradation and occasionally leads to the permanent destruction of soil and loss of fertility [2]. This necessitates the utilization of methods that are environmentally friendly for cleaning up oil spills. One is the use of biological agents which, compared with physicochemical approaches, are better performing and more cost-effective [3]. Bioremediation is feasible, targeted, and capable of achieving high removal efficiency at a low cost.

The biodegradation of crude oil by microorganisms is a “green” alternative for treating hazardous contaminants due to their lack of environmentally degrading effects, as well as the simplicity of their low-energy design, construction, and operation [4,5]. No fungal species exist with the metabolic capability to degrade hydrocarbons of crude oil; thus, the action of fungal consortia is required. A fungal consortium provides a greater spectrum of enzyme activity in bioremoval because it involves the metabolic expression of fungi belonging to each distinct genus [6]. The high capacity to degrade crude oil is also ascribed to many fungal species, such as *Aspergillus*, *Alternaria*, *Cladosporium*, *Eupenicillium*, *Fusarium*, and *Trichoderma* spp. [6,7,8,9].

Nanoparticles can assist microbe activities; however, so far, very few studies have been published regarding the impact of nanoparticles on the microbiological response rates. The higher activity of nanoparticles is usually related to their unique properties and high possible active specific surface areas. Generally, nanoparticle catalysts increase the microbiological reaction rates by positioning themselves on the cells to catalyze the activity of microbes [10,11]. Fungal biodegradation is a complex process [12]. The fungal biodegradation ability of pollutants is significantly affected by several factors, including fungal species, temperature, and hydrocarbon concentration [13]. The response surface method (RSM) is an optimization method for comprehensive test design and mathematical modeling [14]. Little is currently known regarding the molecular characterization of native fungi responsible for degrading crude oil, thus, studies are needed to better understand the utilization of indigenous fungi for in situ bioremediation of crude oil [15].

This study attempted to assess the ability of fungi isolated from the rhizosphere soil of date palms in Riyadh, Saudi Arabia to degrade crude oil under in vitro conditions in the presence of silver nanoparticles. Fungi-degrading crude oil was used to establish a mathematical model using RSM technology, and finally achieve molecular characterization and genetic variation using the inter-simple sequence repeat (ISSR) technique.

## 2. Materials and Methods

### 2.1. Fungal Isolates

Four fungal isolates were isolated in a previous study: *Aspergillus terreus* KC462061, *Aspergillus flavus* AF15, *Trichoderma harzianum* TH07, and *Fusarium solani* FS12. These isolates were identified by internal transcribed spacer (ITS) regions according to the methods of previous studies [16,17].

### 2.2. Synthesis of Silver Nanoparticles (AgNPs)

*A. terreus* KC462061, registered in GenBank as an AgNP producer, was used to synthesize AgNPs. The isolate was supplied by Dr. M. A. Mahmoud, Agriculture Research Center, Giza, Egypt. AgNPs were synthesized using methods described previously [18] to obtain AgNP powder, which was dried through impregnation followed by freeze-drying and H_2_ flow activation according to previously described methods [19].

#### 2.2.1. Transmission Electron Microscopy (TEM)

TEM was performed on a JEOL (JEM-1010) (JEOL: Peabody, MA, USA), with an accelerating voltage of 100 kV after drying a drop of aqueous AgNPs on the carbon-coated copper TEM grid samples, and maintaining under a vacuum in desiccators before loading the specimen holder. The particle size distribution of the AgNPs was evaluated using ImageJ 1.45s software1493, image program developed at the National Institutes of Health and the Laboratory for Optical and Computational Instrumentation (LOCI, University of Wisconsin: Madison, WI, USA).

#### 2.2.2. Scanning Electron Microscopy (SEM)

Scanning electron micrographs were obtained using a JEOL (JSM-6380 LA) (JEOL: Peabody, MA, USA). The samples were filtered and dried prior to SEM.

#### 2.2.3. Energy Dispersive Spectroscopy (EDS)

For EDS, samples were prepared on a copper substrate by the drop coating of AgNPs. Elemental analysis of single particles was carried out using a JEOL (JSM-6380 LA) equipped with a scanning electron microscope.

### 2.3. Crude Oil Degradation by Fungal Isolates

The ability of four fungal isolates to degrade crude oil was evaluated. First, 100 mL of mineral salt medium (MSM) placed in 250 mL flasks was supplemented with 1% crude oil, prepared according to method [20]. The pure fungal isolate (2 mL, 1 × 10^6^ CFU/mL) was inoculated into the MSM. Then, the flasks were incubated at 30 °C and 150 rpm, pH 7.5, for 14 days. At the end of the incubation period, three and seven days, 100 mL of each medium was thoroughly shaken with carbon tetrachloride (50 mL, three times) in a separating funnel, and the three fractions were collected in the case of the crude oil sample. The collected organic layer was dried over anhydrous sodium sulphate. The solvent was removed using a rotary evaporator until a constant weight was achieved. The oil sample was accurately weighed, the percentage of the biodegraded oil was calculated. All treatments, except the sterile control, were performed in triplicate.

#### 2.3.1. Emulsification Activity, E24

To determine the emulsification activity, an equal amount of hexadecane was added to the cell-free culture broth, obtained by centrifugation at 10,000 rpm for 5 min. Samples were incubated for 24 h, and the emulsion index (E24) was calculated according to the following equation [21]:

(E24) = 100 × (height of the emulsion layer/total height)(1)

#### 2.3.2. Surface Tension

Surface tension was measured using the Du Nouy platinum ring method with a Krüss K6 tensiometer (A. Krüss: Hamburg, Germany). The fungal supernatant solution (50 mL) was tested at 25 °C to evaluate the surface tension of bio and chemical surfactants [22]. The value of the surface tension was expressed as mN/m.

#### 2.3.3. Fungal Adhesion to Hydrocarbons (FATH)

The conidial suspension of isolates (6.8 mL) was mixed with 3.2 mL of benzene (Merck) in acid-washed test tubes. The mixture was vortexed for 1 min and following the method of a previous study [23]. Hydrophobicity was calculated as follows:

FATH = initial absorbance − absorbance after phase separation/initial absorbance × 100(2)

### 2.4. Biodegradation of Crude Oil in the Presence of Fungal Isolates and/or AgNPs

This experiment was conducted to assess the impact of two available fungal isolates and/or different concentrations of AgNPs on crude oil biodegradation. The experiment included a fungal isolate (1 × 10^6^ spores/mL) and an aliquot of 2 mL of inoculum, which was inoculated into MSM (100 mL) in a 250 mL Erlenmeyer flask. The cultures were incubated on a temperature-controlled shaker incubator at 150 rpm and 30 °C for seven days using 1 g crude petroleum oil as the sole carbon source. The second experiment consisted of the same previously stated steps, but with only three different concentrations of AgNPs (0.05, 0.1, and 0.2 g). All experiments were performed in triplicates. A control sample was used without the inoculum. Next, flasks were incubated at 30 °C and 150 rpm, pH 7.5, for seven days [24]. The percentage of biodegradation, FATH, surface tension, and emulsification power were determined at days three and seven, and the crude oil samples were extracted from all experiments.

### 2.5. Extraction and Analysis of Crude Oil Degradation by GC-MS

Extraction of crude oil carried out according to pervious method, and alterations in its chemical composition were studied by chromatographic analysis, such as gas chromatography-mass spectrometry (GC-MS). The oil extracted from the biodegraded crude oil was monitored using a Perkin–Elmer GC Clarus 500 system (PerkinElmer: Boston, MA, US) flame ionization detector. Crude oil was separated using a capillary column (30 m × 0.25 mm × 0.25 µm) under the conditions described in a previous study [25]. The inlet temperature program was 50 °C/min. The initial temperature of the oven was maintained at 60 °C for 2 min and increased linearly at a rate of 6 °C/min and was maintained at a final temperature of 300 °C. The operating temperatures of the injector and detector were 300 °C and 320 °C, respectively. The detector temperature was 300 °C. Then, 1 µL of sample was injected with a 1:50 split ratio, and the total run time was less than 35 min.

### 2.6. Identifying Significant Variables Using Box-Behnken Design (BBD)

The biodegradation process was carried out by conducting experiments to determine which variables significantly affected biodegradation efficiency. The BBD for RSM was used to study the combined effect of temperature, pH, and crude oil concentration on the biodegradation of crude oil over three levels. The independent parameters and coding levels of crude oil degradation are shown in Table 1. The independent variables were studied at three different levels: low (−1), medium (0), and high (+1). Crude oil-based MSM was incubated in a shaker at a rate of 160 r/min for seven days and contained as described previously [26]. The absorbance was measured at 225 nm with petroleum ether as a reference. Each sample was measured three times, and the average value was taken.

### 2.7. Genomic DNA Extraction

The fungal isolate spore suspensions (1 × 10^6^ spore/mL) were inoculated into double-layer media in 50 mm Petri dishes, one with liquid peptone yeast glucose (1200 μL), and the other with solid potato dextrose agar as a film. DNA extraction was performed according to a previously described protocol [27].

### 2.8. ISSR-PCR

For ISSR-PCR analysis, a total of 30 primers were synthesized by Intron Biotechnology, South Korea according to the primer sequences [28]. The ISSR amplification reactions were performed as previously described [28,29,30]. The ISSR PCR mixture were carried out in volumes of 25 µL containing 2.5 µL 10× buffer (with 15 mmol/L MgCl2), 2 µL (15–20 ng) of template DNA, 2 µL primer (10 pmole/µL), 2.0 µL of dNTPs (2.5 mmol/L each), 0.5 µL of Taq DNA polymerase (5U/µL; BioLabs, United Kingdom) and 16 µL Sterile water.

PCR products were detected with 1.5% agarose ethidium bromide gels in Tris-acetate-EDTA (TAE) 1× buffer (40 mM Tris-acetate and 1.0 mM Ethylenediamine tetraacetic acid (EDTA). A 100 bp DNA ladder (Intron Biotechnology: Gyeonggi-do, Korea) was used as the molecular marker.

Polymorphism (%) was calculated as follows:

Polymorphism % = (no. of polymorphic band/total number of bands) × 100(3)

### 2.9. Data Analysis

Statistical analysis was done using SPSS (version 15,IBM SPSS Statistics, International Business Machines Corporation, Armonk, NY, USA) was used to assess the ability to utilize petroleum hydrocarbons among the 4 fungal isolates based on their abilities. A randomized complete block design was used in the present study. The standard deviation is calculated by SPSS using the descriptive procedure.

## 3. Results and Discussion

### 3.1. Molecular Identification

The morphological and microscopic fungal isolate characteristics and molecular tools used for isolate identification were compared with those of reference isolates in the NCBI GenBank. These four isolates were classified as *A. terreus* KC462061, *A. flavus* AF15, *T. harzianum* TH07, and *F. solani* FS12, as shown in Table 2. In the BLAST-based analysis, the ITS region sequence of each identified fungal isolate showed that isolate *A. terreus* KC462061 was 99% similar to *A. terreus* GU 966497 (Figure S1). Using the same method, *A. flavus* AF15 showed 98% similarity with *A. flavus* KY488467 (Figure S2), and *T. harzianum* TH07 showed 99% similarity with *T. harzianum* KC569346 (Figure S3), while *F. solani* FS12 showed 98% similarity with *F. solani* MF136402 (Figure S4).

The sequence of the ITS1-5.8S-ITS2 region appeared to be a credible molecular approach for fungal identification and discrimination [30]. The ITS region is able to differentiate and provide accurate and rapid identification of many fungi genera, such as *Aspergillus*, *Fusarium*, and *Trichoderma* at the species level [31].

### 3.2. Characterization of Fungal AgNPs

**Figure 1:**
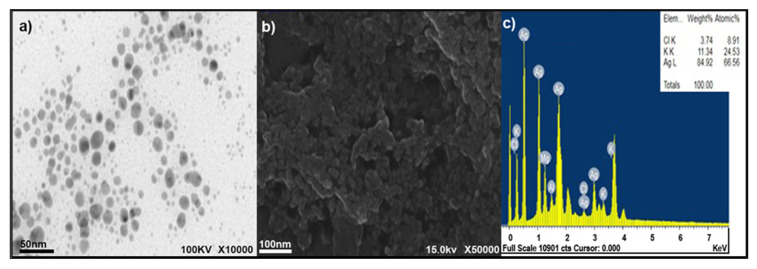
Characterization of fungal silver nanoparticles AgNPs (**a**) TEM micrograph of biosynthesized AgNPs, (**b**) SEM micrograph of biosynthesized AgNPs, and (**c**) energy dispersive spectroscopy EDS micrograph of biosynthesized silver AgNPs.

AgNPs were examined using TEM, SEM, and EDS. Figure 1a illustrates the TEM micrograph of the biosynthesized AgNPs. The TEM micrograph shows that the as-synthesized NPs had a spherical morphology, in which nanoparticles were not conglomerated. The particle size ranged from 35 to 60 nm. TEM imaging is an essential method for observing the formation and stabilization of AgNPs.

The size and shape of the biosynthesized nanoparticles appear to depend on the type of microorganisms and other factors, such as temperature and pH of the medium, thus, a considerable size variability in AgNPs is produced by different *Aspergillus* spp. [32].

The SEM image of the AgNPs is shown in Figure 1b. The SEM micrograph of the AgNPs did not show a uniform surface but had three dimensions. The nanoparticles tended to be oval to spherical in shape. Most of the nanoparticles were aggregated, and no individual particles were present. Several AgNPs clusters were present, which could be attributed to the aggregation of NPs formed throughout specimen preparation [33].

Furthermore, the elemental structure of the synthesized specimen was also measured by EDX analysis (Figure 1c), showing the formation of pure AgNPs. However, the mass present was approximately 85% of the sample. The EDS profile also showed a strong silver signal along with weak carbon peaks, strongly suggesting that Ag was the major element. Clear identification of the synthesized AgNPs of the elemental composition profile was achieved. The intense signal at 3 keV strongly suggested that Ag was the main element showing optical absorption in this range due to the resonance of the surface plasmon [34].

### 3.3. Crude Oil Degradation by Fungal Isolates

During the fungal degradation of crude oil, the investigated fungal isolates showed perfect levels of crude oil biodegradation ability (Table 3). *A. flavus* AF15 showed the maximum biodegradation ability of 41.94%, followed by *T. harzianum* TH07 (37.36%), while third-ranking was *Aspergillus terreus* KC462061 with moderate biodegradation ability whereas the minimum biodegradation ability (19.42%) was recorded for *F. solani* FS12. In addition to the surface tension, FATH, and emulsification activity, assays were also performed for the four fungal isolates (Table 3). *A. flavus* AF15 had the best physicochemical properties with high emulsification activity (E24; 51.57%) and low FATH (36.74%) and surface tension (39.80 N/m), followed by *T. harzianum* TH07.

*Aspergillus oryzae* KR029081 decreased the surface tension to 23 N/m, had higher emulsification activity (E24; 75.6%), and showed a decrease in FATH (26.5%). This indicates a very close correlation between some physical properties and crude oil degradation by fungi [35]. *Penicillium* spp. KY883662 reduced the surface tension and exhibited a cell surface hydrophobicity of more than 70%. Furthermore, it showed effective crude oil-degrading activity and crude oil emulsification [36].

### 3.4. Biodegradation of Crude Oil by Two Fungal Isolates and/or AgNPs

In the present study, two fungal isolates (*A. flavus* AF15 and *T. harzianum* TH07) were grown together or separately with/without three different concentrations of AgNPs, and three different concentrations of AgNPs were used separately. The biodegradation percentage of crude oil is listed in Table 4. Some factors related to biodegradation were assayed after three and seven days. The results showed that all treatments utilized for the degradation of crude oil were potential degraders, with a biodegradation percentage ranging from 12.4% to 57.8% after seven days. The highest extent of biodegradation was 57.8% with a combination of *A. flavus* AF15 and *T. harzianum* TH07, and 0.05 g AgNPs. The degradation level was very slight when three different concentrations of AgNPs were used separately, ranging from 12.4% to 15.1% after seven days.

The fungal biodegradation of aromatic hydrocarbons mechanisms is generally related to detoxification and bioremediation by many enzymes. Three very different—yet not mutually exclusive—enzymatic mechanisms have been proposed: (1) oxidation of the aromatic ring by intracellular Cytochromes P450 monooxygenases (CYP monooxygenases) for detoxification, (2) the nonspecific and coincidental oxidative action of extracellular aromatics by excreted lignin-degrading peroxidases, (3) the same kind of coincidental action involving extracellular laccases [37]. Our results were similar to [38] who isolated crude oil-degrading fungal strain, *Aspergillus* sp. RFC-1 (Rumaila field oil in Basra, Iraq); they reduced surface tension and also found an inverse relationship between potent the crude oil biodegradation of fungi and a decrease in surface tension. Correlation of Fungal Biodegradation and crude oil depends on fungi increasing emulsification activity and reduced surface tension, increasing the fungal growth rate on hydrocarbons. Fungi also play a critical role in modifying cell surface hydrophobicity. Fungal isolates can act as emulsifying agents by decreasing surface tension, improving crude oil biodegradation through two mechanisms. The first mechanism promotes the emulsification of hydrophobic compounds to mycelial pellet structures. The second mechanism induces high cell surface hydrophobicity, thus increasing the direct physical contact between cells and poor water-soluble substrates [38,39].

Furthermore, fungal consortia play a key role as highly efficient agents for the bioremediation of contaminated environments [40]. A consortium of an enzyme laccase, manganese peroxidase (MnP), Lignin peroxidase (LiP), and catalase showed a significant level of enzyme activity compared to a solo enzyme. A fungal consortium provides a greater spectrum of enzymes compared to an individual fungus, and remarkably higher enzyme activity due to the synergistic effect of the pooled genotypes. Moreover, it establishes very effective bioremediation agents [6]. Most Basidiomycete fungi are capable of producing extracellular ligninolytic enzymes (laccase, manganese peroxidase, lignin peroxidase, and versatile peroxidase) and accessory enzymes (H_2_O_2_-generating enzymes), both of which are responsible for the degradation of lignin. Because of the nonspecific nature of an extracellular fungal ligninolytic system, this could be a useful and powerful tool for bioremediation purposes [41]. The degradation percentage of crude oil by Bacillus licheniformis was up to 60% when the medium contained specific concentrations of Fe_2_O_3_ and Zn_5_OH_8_Cl_2_ nanoparticles and biosurfactant [42]. Genetic modification technologies (GM) and genome-wide (-omics) technologies are available that can act as enabling technologies, so-called ‘ecogenomics’, to develop bioremediation in the field. Ecogenomic approaches could be used to characterize contaminated sites and monitor the bioremediation process [37].

### 3.5. Analysis of Crude Oil Biodegradation by GC-MS

**Figure 2:**
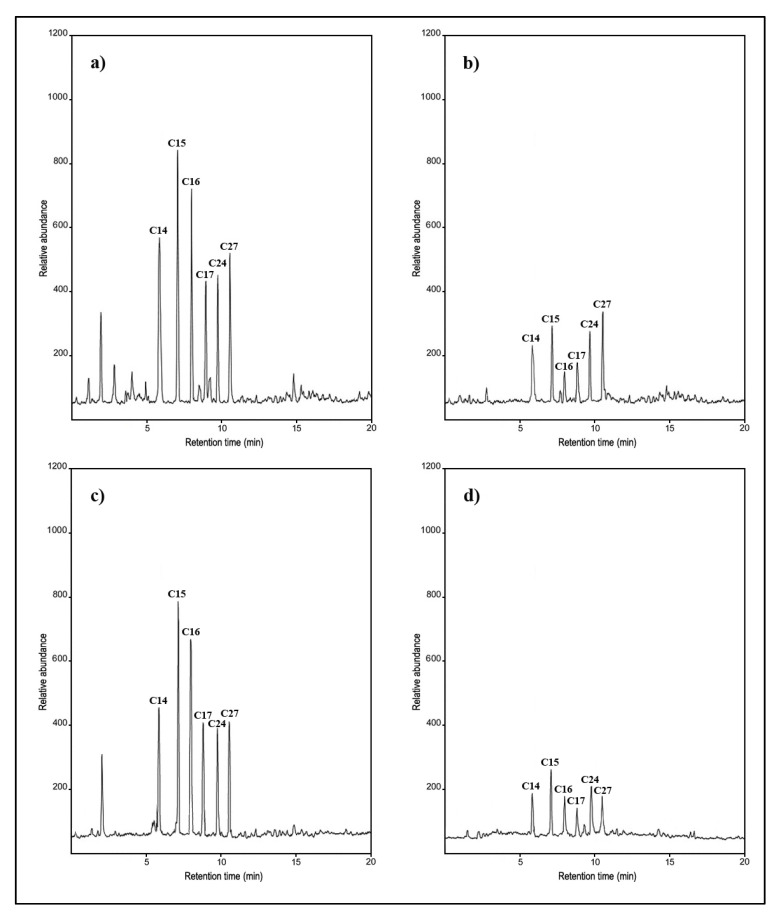
Gas chromatography-mass spectrometry (GC-MS) analysis of crude oil samples before and after biodegradation. (**a**) The crude oil control; (**b**) biodegradation sample of *A. flavus* AF15 and AgNPs 0.05 g; (**c**) degradation sample of AgNPs 0.2 g and (**d**) degradation sample of *A. flavus* AF15, *T. harzianum* TH07 and AgNPs 0.05 g, all treatments after 7 days.

GC-MS of the crude oil treated by the fungal consortium, AgNPs, and control samples of crude oil are shown in Figure 2. From this figure, it can be observed that the fungal consortium and/or AgNPs behaved differently in the degradation of crude oil. Figure 2a shows the crude oil control with the main hydrocarbon compound peaks of C_14_, C_15_, C_16_, C_17_, C_24_, and C_27_, which were identified at retention times of 6.250, 6.945, 7.700, 8.350, 9.400, and 10.450 min by comparing the data with standard library compounds. All the hydrocarbons present in the crude oil identified using GC-MS analysis were effectively degraded by different treatments containing fungal consortia and AgNPs. Figure 2b displays a good biodegradation percentage (53.4%) when *A. flavus* AF15 combined with 0.05 g AgNPs. Figure 2c appears to show a weak biodegradation percentage when high concentrations of AgNPs (0.2 g) were used to separately express the negative effect of AgNPs. Figure 2d shows the highest biodegradation percentage (57.8%) when a fungal consortium consisting of *A. flavus* AF15 and *T. Harzianum* TH07 and AgNPs 0.05 g was used, and the positive effect of the AgNPs was apparent.

GC-MS is a highly accurate, effective, and versatile analytical technique with numerous scientific applications. GC-MS has become a highly recommended tool for monitoring and tracking hydrocarbons and chemical pollutants in the environment. It can be used to screen the degradation products of hydrocarbons, even C_60_ [43]. Using GC-MS data to analyze the biodegradation of crude oil by microbial consortium, it was found to be strongly degraded with a degradation ratio of up to 56% after four weeks of treatment [6]. GC-MS analysis showed that the *A. oryzae* KR029081 isolate was very unique and degraded with a hydrocarbon from C_14_ to C_27_ with biodegradation of 99% [35].

### 3.6. Optimization of Biodegradation Using RSM

Five significant variables were optimized using the BBD (Table 5). Table 5 shows good agreement between the predicted and experimental values. A total of 30 experiments were performed with a different combination of five variables. The maximum biodegradation of crude oil (57.21%) was achieved in run number 16 under the following conditions: temperature of 30 °C, pH 7, crude oil concentration of 4 g/L, 1:1 ratio between *A. flavus* AF15 and *T. harzianum* TH07, and AgNP concentration of 0.05 g. Analysis of regression coefficients and *t*-value of five variables are presented in Table 6. The results showed that temperature (confidence level, 99.81%), pH (99.66%), crude oil concentration (99.52%), ratio between *A. flavus* AF15 and *T. harzianum* TH07 (96.18%), and AgNP concentration (95.79%) were the most significant factors influencing biodegradation efficiency.

The three most important factors were temperature, pH, and AgNPs concentration. These factors were used to establish a mathematical model using RSM technology. The mathematical model has optimized conditions of biodegradation utilizing these factors, and the best degradation rate was 81.63% with a temperature of 30 °C, pH of 7.14, and TPH concentration of 4.83 g/L [26].

### 3.7. Molecular Characterization of Fungal Isolates by ISSR

#### 3.7.1. *A. flavus* Isolates

**Figure 3:**
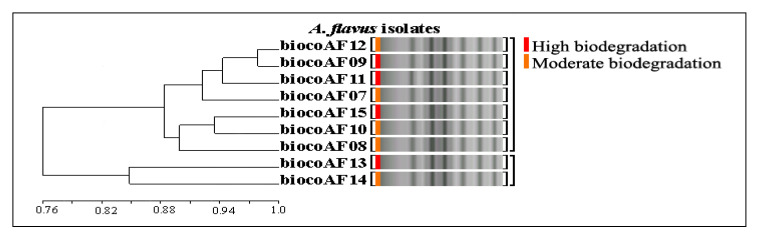
UPGMA dendrogram based on Jaccard’s coefficient illustrating the genetic similarities among nine *A. flavus* isolates based on (GA)_8_C primer.

**Figure 4:**
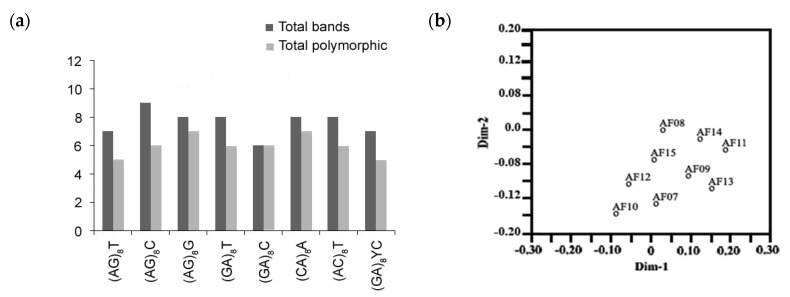
(**a**) Bar graph for nine *A. flavus* showing various fragments produced by primers and total polymorphisms produced by them, (**b**), 2D principal component analysis (PCA) based on genetic relationships from inter-simple sequence repeat (ISSR) data using the DECENTER and EIGEN features of Numerical Taxonomy and Multivariate Analysis System (NTSYSpc).

Of the 15 ISSR primers, nine showed reproducible and polymorphic DNA amplification patterns. A total of 53 DNA bands were obtained with an average of 5.8 DNA bands per primer, and 42 DNA bands (79.2%) were polymorphic bands with an average of 4.6 polymorphic DNA bands per primer. Genetic similarity ranged from 0.76 (Figure 3). The (GA)_8_C primer showed 100% polymorphism (Figure 4a), followed by (AG)_8_G, then (CA)_8_A (87.5%), and finally (AG)_8_C (66.6%). A two-dimensional principal component analysis (PCA) presenting the relationship of nine ISSR markers based on unweighted pair group method with arithmetic mean (UPGMA) cluster analysis showed that the nine *A. flavus* isolate populations were similar, classifying them into one group (Figure 4b).

#### 3.7.2. *T. harzianum* Isolates

**Figure 5:**
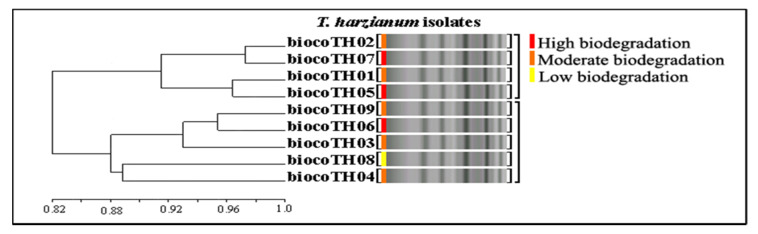
UPGMA dendrogram based on Jaccard’s coefficient illustrating the genetic similarities among nine *T. harzianum* isolates based on (GA)_8_T primer.

**Figure 6:**
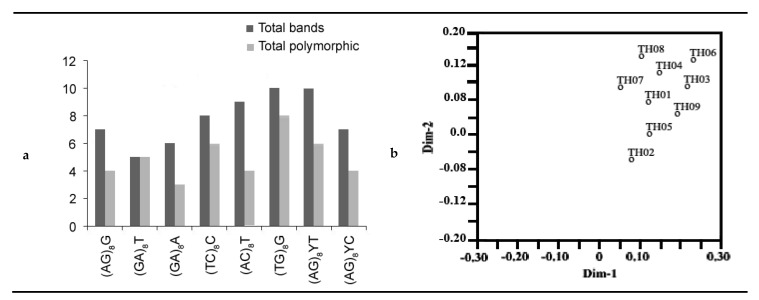
(**a**) Bar graph for nine *T. harzianum* showing various fragments produced by primers and total polymorphisms produced by them, (**b**), 2D principal component analysis (PCA) based on genetic relationships from ISSR data using the DECENTER and EIGEN features of NTSYSpc.

Of the 15 primers used, nine produced unambiguous fragments with repeatable patterns. 62 DNA bands were obtained with an average of 6.8 DNA bands per primer, and 40 DNA bands (64.5%) were polymorphic bands with an average of 4.4 polymorphic DNA bands per primer. The genetic similarity coefficient ranged from 0.82 to 0.95 Figure 5. The highest number of bands (10) was obtained with two primers, (TG)_8_G and (AG)_8_YT, while the lowest number five was obtained with primer (GA)_8_T (Figure 6a). Only one primer, (GA)_8_T, had good polymorphism (100%), and other polymorphic primers exhibited low values of polymorphism, ranging from 44.5% to 80% (Figure 6a). The genetic relationships among the nine *T. harzianum* isolates were also visualized by 2D PCA for nine ISSR primers (Figure 6b).

ISSR-PCR is a highly effective method for the characterization of *Aspergillus* and *Trichoderma* spp., and has also been shown to be useful for assessing the genetic diversity of two species. Moreover, it has been proven to be useful for DNA fingerprinting using ISSR genetic markers [28,44].

## 4. Conclusions

The two fungal isolates used in this study demonstrated rapid crude oil biodegradation ability and when used together as a consortium displayed a cooperative effect that enhanced the biodegradation process. In addition, we have shown the positive effect of low concentrations of AgNPs on biodegradation. RSM is an efficient mathematical method to optimize the microbial biodegradation of crude oil. ISSR-PCR is a highly effective method for the characterization of fungi responsible for biodegrading crude oil.

## Figures and Tables

**Table 1:** Levels of variables for the experimental design.

Independent Variables	Level		
	−1	0	1
Temperature, °C	25	30	35
pH	6	7	8
Crude oil concentration, g/L	2	4	6
Ratio between *A. flavus* AF15 and *T. harzianum* TH07	1:2	1:1	2:1
AgNPs concentration g	0.05	0.1	0.2

**Table 2:** Identification of soil-borne fungi species by sequencing of Internal Transcribed Spacer1 (ITS1) and ITS 2 and region of 5.8S rRNA gene compared with sequences listed in the GenBank.

Fungi of Soil		Fungi of GenBank		Identity
Isolate Name	Accession No.	Name	Accession Number	
*A.* *terreus*	KC462061 *	*A.* *terreus*	GU966497	99%
*A.* *flavus*	AF15 **	*A.* *flavus*	KY488467	98%
*T.* *harzianum*	TH07 **	*T.* *harzianum*	KC569346	99%
*F.* *solani*	FS12 **	*F.* *solani*	MF136402	98%

* Accession no. by genbank, ** Accession no. by authors.

**Table 3:** Mean biodegradation percentage for four isolates, surface tension, cell surface hydrophobicity, emulsification activity after two weeks of incubation at 30 °C.

Fungal Isolates	Mean of % Biodegradation	Surface Tension (mN/m)	Cell Surface Hydrophobicity (FATH, %)	Emulsification Activity (E24; %)
*A. terreus* KC462061	23.87 ± 0.05	52.31 ± 0.20	46.58 ± 0.72	41.26 ± 0.52
*A. flavus* AF15	41.94 ± 0.76	39.80 ± 0.57	36.74 ± 0.64	51.57 ± 0.38
*T. harzianum* TH07	37.36 ± 0.49	43.21 ± 0.82	39.51 ± 0.55	47.23 ± 0.31
*F. solani* FS12	19.42 ± 0.81	53.92 ± 0.14	49.74 ± 0.21	44.81 ± 0.64

Note: The surface tension of Bushnell–Haas medium without fungi was 61.7 mN/m. Data are mean values with standard deviation (±SD).

**Table 4:** Percentage of bioremediation some physicochemical properties of crude oil after treatment with *A. flavus* AF15 or/and *T. harzianum* TH07 using different concentrations of AgNPs.

Sample	Incubation Periods (Days)			Percentage of Biodegradation ^a^ (%)	Incubation Periods (Days)			Percentage of Biodegradation ^a^ (%)
	After 3 Days				After 7 Days			
	Surface Tension (mN/m)	Cell Surface Hydrophobicity (FATH, %)	Emulsification Activity (E24; %)		Surface Tension (mN/m)	Cell Surface Hydrophobicity (FATH, %)	Emulsification Activity (E24; %)	
crude oil (Control)	62.4	0.0	0.0	0.00	62.4	0.0	0.0	0.00
Crude oil + AF	42.7	41.5	54.5	17.2	38.6	40.7	46.1	45.5
Crude oil + AF + AgNPs 0.05 g	47.1	45.7	59.1	25.6	35.4	43.2	61.4	53.4
Crude oil + AF + AgNPs 0.1 g	45.5	47.2	55.4	22.5	42.8	42.3	57.2	50.6
Crude oil + AF + AgNPs 0.2 g	46.2	42.3	54.7	21.3	45.2	45.8	55.7	49.3
Crude oil + TH	49.9	46.9	57.3	18.9	40.9	51.9	42.6	42.8
Crude oil + TH + AgNPs 0.05 g	52.3	44.7	54.9	23.4	34.5	53.2	64.2	51.5
Crude oil + TH + AgNPs 0.1 g	49.1	44.8	51.2	24.3	39.7	57.5	59.3	48.5
Crude oil + TH + AgNPs 0.2 g	48.5	45.3	49.8	21.7	45.3	52.5	56.8	46.2
Crude oil + AF + TH	39.7	43.1	51.4	29.4	35.2	59.6	65.2	49.3
Crude oil + AF + TH + AgNPs 0.05 g	40.4	41.9	63.1	31.6	32.8	63.4	67.9	57.8
Crude oil + AF + TH + AgNPs 0.1 g	40.1	43.4	59.7	28.9	36.1	63.1	64.5	53.7
Crude oil + AF + TH + AgNPs 0.2 g	38.5	44.6	57.9	25.5	38.7	59.3	60.2	50.4
Crude oil + AgNPs 0.05 g	51.3	49.3	31.4	13.8	49.6	29.7	35.1	15.1
Crude oil + AgNPs 0.1 g	52.9	48.6	28.2	11.7	50.2	31.4	33.7	13.9
Crude oil + AgNPs 0.2 g	54.8	46.1	27.5	9.2	53.9	32.7	32.9	12.4

^a^ Percentage biodegradation = Weight of original oil—wt. of residual/wt. of original oil.

**Table 5:** Full-factorial Box-Behnken design matrix of 5 independent variables and mean response for biodegradation percentage of crude oil.

Run	Temperature	pH	Crude Oil Conc.	Ratio between AF and TH	Nano Conc.	Biodegradation (%)	
						Actual	Predicted
1	0	−1	−1	−1	−1	45.69	48.17
2	0	0	0	0	0	49.27	58.81
3	0	1	1	1	1	27.53	28.82
4	0	0	−1	−1	−1	35.91	37.80
5	0	0	0	−1	−1	38.11	40.85
6	1	−1	−1	−1	−1	38.13	39.71
7	1	0	0	0	0	43.67	44.92
8	1	1	1	1	1	31.83	20.58
9	1	0	−1	−1	−1	33.66	30.58
10	1	0	0	−1	−1	35.19	37.44
11	−1	−1	−1	−1	−1	37.12	34.91
12	−1	0	0	0	0	36.67	38.26
13	−1	1	1	1	1	21.56	19.71
14	−1	0	−1	−1	−1	32.24	34.78
15	−1	0	0	−1	−1	28.35	25.57
16	0	0	0	0	−1	57.21	59.16
17	0	1	−1	−1	−1	20.62	22.37
18	0	1	0	0	0	32.85	35.72
19	0	1	−1	0	0	34.78	36.55
20	0	1	−1	−1	0	29.51	27.93
21	1	0	0	0	−1	42.26	44.71
22	1	1	−1	−1	−1	32.55	34.78
23	1	1	0	0	0	29.14	31.03
24	1	1	−1	0	0	34.71	35.98
25	1	1	−1	−1	0	41.63	39.39
26	−1	0	0	0	−1	44.15	42.92
27	−1	1	−1	−1	−1	37.42	40.07
28	−1	1	0	0	0	35.11	32.55
29	−1	1	−1	0	0	32.84	35.23
30	−1	1	−1	−1	0	28.17	30.83

**Table 6:** Estimated regression coefficients for optimization of biodegradation efficiency using Box-Behnken design.

Variables	Main Effect	*t*-Value	*p*-Value	Confidence Level (%)
Temperature, °C	0.49	14.72	0.000	99.81
pH	0.52	11.34	0.000	99.66
Crude oil concentration, g/L	0.44	12.68	0.000	99.52
Ratio between AF and TH	0.23	6.53	0.024	96.18
AgNPs concentration	0.17	7.16	0.031	95.79

## Supplementary Materials

The following are available online at https://www.mdpi.com/1660-4601/17/18/6564/s1, Figure S1: Phylogenetic tree of *A. terreus* biocoAT (KC462061) based on ITS region and 5.8S sequences, Figure S2: Phylogenetic tree of *A. flavus* biocoAF15 based on ITS region and 5.8S sequences, Figure S3: Phylogenetic tree of *T. harzianum* biocoTH07 based on ITS region and 5.8S sequences, Figure S4: Phylogenetic tree of *F. solani* biocoFS12 based on ITS region and 5.8S sequences.
