# Supplementary material for: Bioremediation of Crude Oil by Rhizosphere Fungal Isolates in the Presence of Silver Nanoparticles
Source: Int J Environ Res Public Health. 2020 Sep 9;17(18):6564. doi: 10.3390/ijerph17186564 (PMC7560104; doi:10.3390/ijerph17186564)
Supplement: Supplementary file 1 [file ijerph-17-06564-s001.pdf]

International Journal of
Environmental Research and Public Health

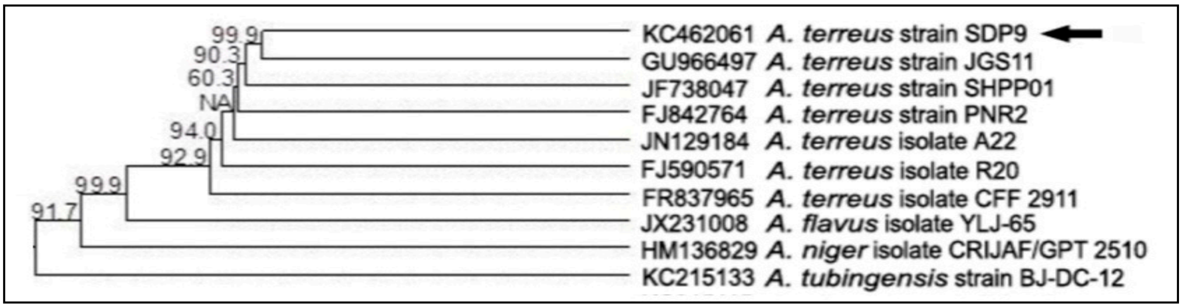

Figure S1. Phylogenetic tree of *A. terreus* biocoAT (KC462061) based on ITS region and 5.8S sequences.

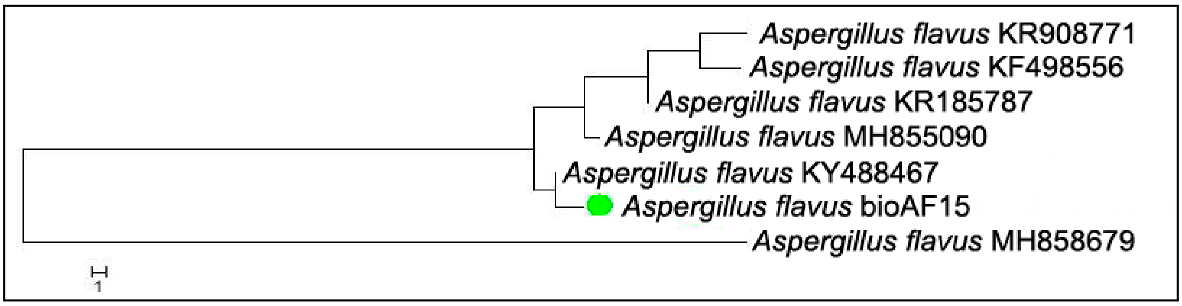

Figure S2. Phylogenetic tree of *A. flavus* biocoAF15 based on ITS region and 5.8S sequences.

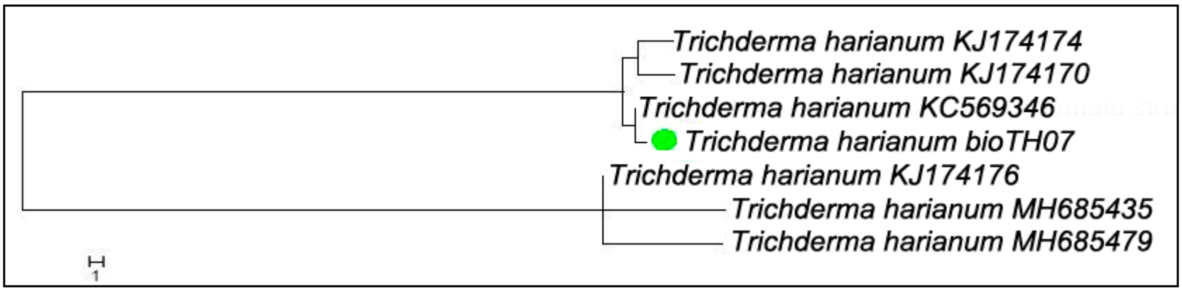

Figure S3. Phylogenetic tree of *T. harzianum* biocoTH07 based on ITS region and 5.8S sequences.

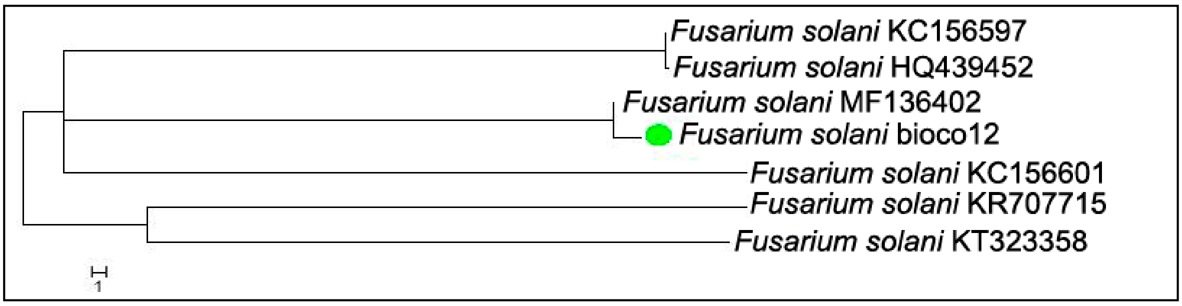

Figure S4. Phylogenetic tree of *F. solani* biocoFS12 based on ITS region and 5.8S sequences.
